# Supplementary material for: Charting cellular differentiation trajectories with Ricci flow
Source: Nat Commun. 2024 Mar 13;15:2258. doi: 10.1038/s41467-024-45889-6 (PMC10937996; doi:10.1038/s41467-024-45889-6)
Supplement: Supplementary file 2 — Reporting Summary [file 41467_2024_45889_MOESM2_ESM.pdf]

nature portfolio

Corresponding author(s): Anthony Baptista and Christopher R. S. Banerji

Last updated by author(s): Jan 4, 2024

Reporting Summary

Nature Portfolio wishes to improve the reproducibility of the work that we publish. This form provides structure for consistency and transparency in reporting. For further information on Nature Portfolio policies, see our Editorial Policies and the Editorial Policy Checklist.

Statistics

For all statistical analyses, confirm that the following items are present in the figure legend, table legend, main text, or Methods section.

n/a	Confirmed	
☐	☒	The exact sample size (*n*) for each experimental group/condition, given as a discrete number and unit of measurement
☒	☐	A statement on whether measurements were taken from distinct samples or whether the same sample was measured repeatedly
☐	☒	The statistical test(s) used AND whether they are one- or two-sided<br>*Only common tests should be described solely by name; describe more complex techniques in the Methods section.*
☐	☒	A description of all covariates tested
☐	☒	A description of any assumptions or corrections, such as tests of normality and adjustment for multiple comparisons
☐	☒	A full description of the statistical parameters including central tendency (e.g. means) or other basic estimates (e.g. regression coefficient) AND variation (e.g. standard deviation) or associated estimates of uncertainty (e.g. confidence intervals)
☐	☒	For null hypothesis testing, the test statistic (e.g. *F*, *t*, *r*) with confidence intervals, effect sizes, degrees of freedom and *P* value noted<br>*Give P values as exact values whenever suitable.*
☒	☐	For Bayesian analysis, information on the choice of priors and Markov chain Monte Carlo settings
☒	☐	For hierarchical and complex designs, identification of the appropriate level for tests and full reporting of outcomes
☐	☒	Estimates of effect sizes (e.g. Cohen's *d*, Pearson's *r*), indicating how they were calculated

Our web collection on statistics for biologists contains articles on many of the points above.

Software and code

Policy information about availability of computer code

Data collection	No software was used for data collection
Data analysis	Network entropy was computed using the SCENT package in R available on GitHub https://github.com/aet21/SCENT, using the network provided at https://github.com/aet21/SCENT/blob/master/data/net17Jan16.rda.<br><br>The R code developed for the analysis presented in the paper for computing Forman-Ricci curvature, evaluating Ricci flow and comparing to straight line trajectories is accessible in the following Github: https://github.com/anthbapt/Cellular-differentiation-trajectories-with-Ricci-flow. The version of the code used is associated with the following DOI: 10.5281/zenodo.10469562.<br>All code was implemented using R version 4.2.2.

For manuscripts utilizing custom algorithms or software that are central to the research but not yet described in published literature, software must be made available to editors and reviewers. We strongly encourage code deposition in a community repository (e.g. GitHub). See the Nature Portfolio guidelines for submitting code & software for further information.

Data

Policy information about availability of data

All manuscripts must include a data availability statement. This statement should provide the following information, where applicable:

- Accession codes, unique identifiers, or web links for publicly available datasets
- A description of any restrictions on data availability
- For clinical datasets or third party data, please ensure that the statement adheres to our policy

Normalised read count data corresponding to RNA-sequencing data was downloaded from GEO \cite{Barrett2012}. Data from \cite{chu2016} describing scRNAseq of 1018 single cells assayed at different stages of multipotency and alongside data describing 758 single cells assayed at 6 distinct time points during ESC differentiation was downloaded from accession GSE75748. Data from \cite{Tirosh2016} describing scRNAseq of 1257 malignant and 3256 healthy single cells from 19 patients with malignant melanoma was downloaded from accession GSE72056. Data from \cite{Li2017} describing scRNAseq of 272 malignant and 160 healthy cells from patients with colorectal cancer was downloaded from accession GSE81861. Our data set describing healthy myoblast differentiation at 8 distinct time points was downloaded from accessions GSE102812 and GSE123468 \cite{Banerji2018}.

Research involving human participants, their data, or biological material

Policy information about studies with human participants or human data. See also policy information about sex, gender (identity/presentation), and sexual orientation and race, ethnicity and racism.

Reporting on sex and gender	N/A - all data used was synthetic or obtained from public repository (GEO) as above
Reporting on race, ethnicity, or other socially relevant groupings	N/A
Population characteristics	N/A
Recruitment	N/A
Ethics oversight	N/A

Note that full information on the approval of the study protocol must also be provided in the manuscript.

Field-specific reporting

Please select the one below that is the best fit for your research. If you are not sure, read the appropriate sections before making your selection.

☒ Life sciences ☐ Behavioural & social sciences ☐ Ecological, evolutionary & environmental sciences

For a reference copy of the document with all sections, see nature.com/documents/nr-reporting-summary-flat.pdf

Life sciences study design

All studies must disclose on these points even when the disclosure is negative.

Sample size	Data from [42] describing scRNAseq of 1018 single cells assayed at different stages of multipotency and alongside data describing 758 single cells assayed at 6 distinct time points during ESC differentiation was downloaded from accession GSE75748. Data from [43] describing scRNAseq of 1257 malignant and 3256 healthy single cells from 19 patients with malignant melanoma was downloaded from accession GSE72056. Data from [44] describing scRNAseq of 272 malignant and 160 healthy cells from patients with colorectal cancer was downloaded from accession GSE81861. Our data set describing healthy myoblast differentiation at 8 distinct time points was downloaded from accessions GSE102812 and GSE123468 [45]. Total transcriptomic sample count: 6745. Sample size calculation was not performed as this was an exploratory study of novel network measures and effect size could not be estimated a prori, however the sample count used was in excess of those used in previous published works exploring similar constructs (e.g., [22] &[25]) and we analysed all data sets considered in these previous studies to provide comparison.
Data exclusions	No data was excluded
Replication	Each of the 5 public GEO studies described above contain replication of samples, at the very least as triplicates.
Randomization	No randomisation was required for our analysis of public data, this public data was not collected by the us and all data published was analysed. Our study investigated two network measures in a wide range of biological cells obtained from 4 independent studies and their association with phenotype. There was no treatment/intervention requiring randomisation.
Blinding	No blinding was required for our analysis of public data, this data was not collected by us and and all data published was analysed. Our study investigated two network measures in a wide range of biological cells obtained from 4 independent studies and their association with phenotype. There was no treatment/intervention requiring blinding.

Reporting for specific materials, systems and methods

We require information from authors about some types of materials, experimental systems and methods used in many studies. Here, indicate whether each material, system or method listed is relevant to your study. If you are not sure if a list item applies to your research, read the appropriate section before selecting a response.

Materials & experimental systems

n/a	Involved in the study
☒	☐ Antibodies
☒	☐ Eukaryotic cell lines
☒	☐ Palaeontology and archaeology
☒	☐ Animals and other organisms
☒	☐ Clinical data
☒	☐ Dual use research of concern
☒	☐ Plants

Methods

n/a	Involved in the study
☒	☐ ChIP-seq
☒	☐ Flow cytometry
☒	☐ MRI-based neuroimaging

Plants

Seed stocks	N/A - all data used was synthetic or obtained from public repository (GEO) as above
Novel plant genotypes	N/A
Authentication	N/A
